# NMR ^1^H,^19^F-based screening of the four stem-looped structure 5_SL1–SL4 located in the 5′-untranslated region of SARS-CoV 2 RNA†

**DOI:** 10.1039/d3md00322a

**Published:** 2023-11-28

**Authors:** Daniel Hymon, Jason Martins, Christian Richter, Sridhar Sreeramulu, Anna Wacker, Jan Ferner, Neeraj N. Patwardhan, Amanda E. Hargrove, Harald Schwalbe

**Affiliations:** a Institute for Organic Chemistry and Chemical Biology, Center for Biomolecular Magnetic Resonance (BMRZ), Goethe-University Frankfurt Max-von-Laue-Str. 7 60438 Frankfurt/Main Germany schwalbe@nmr.uni-frankfurt.de; b Department of Chemistry, Duke University Durham North Carolina 27708 USA

## Abstract

Development of new antiviral medication against the beta-coronavirus SARS-CoV-2 (SCoV2) is actively being pursued. Both NMR spectroscopy and crystallography as structural screening technologies have been utilised to screen the viral proteome for binding to fragment libraries. Here, we report on NMR screening of elements of the viral RNA genome with two different ligand libraries using ^1^H-NMR-screening experiments and ^1^H and ^19^F NMR-screening experiments for fluorinated compounds. We screened against the 5′-terminal 119 nucleotides located in the 5′-untranslated region of the RNA genome of SCoV2 and further dissected the four stem-loops into its constituent RNA elements to test specificity of binding of ligands to shorter and longer viral RNA stretches. The first library (DRTL-F library) is enriched in ligands binding to RNA motifs, while the second library (DSI-poised library) represents a fragment library originally designed for protein screening. Conducting screens with two different libraries allows us to compare different NMR screening methodologies, describe NMR screening workflows, validate the two different fragment libraries, and derive initial leads for further downstream medicinal chemistry optimisation.

## Abstract 2

Exploring the benefits and limits of ligand based combined ^1^H and ^19^F NMR readout for interaction studies of viral RNAs with small compounds.

## Graphical abstract

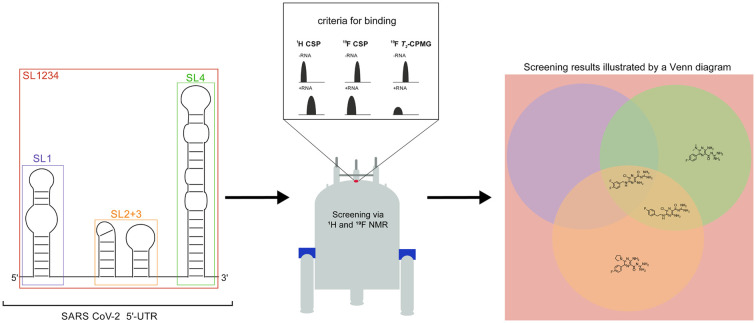

## Introduction

The global pandemic caused by SARS-CoV-2 (ScoV2) has seen the successful use of vaccines but also challenges for their efficacy against the variants of concern that have emerged during viral evolution.^1^ The first-generation oral therapeutics, molnupiravir and the combinational drug Paxlovid (nirmatrelvir (also known as PF-07321332) plus ritonavir) were initially heralded as revolutionary until clinical-trial data revealed lower-than-expected efficacy.^2–4^ Therefore, continued efforts in developing new antivirals not only against the validated viral targets (proteases and polymerases) but also exploring other targets are required to evade resistance.^5^

The virus contains a small proteome of 27 proteins and an RNA genome of a little less than 30.000 nucleotides. Most efforts to develop antiviral drugs focus on targeting proteins, because some viral proteins have been classified as validated targets, and technologies, including fragment screening by X-ray crystallography, have been established.^6–10^ By stark contrast, only recently, targeting antiviral RNA has gained interest.^11–15^ In fact, targeting conserved structural motifs of viral RNAs with molecules of low molecular weight (small molecules) would provide vast opportunities in developing alternative approaches to inhibit viral proliferation.^16^ Such targeting of RNAs by small molecules is not only relevant towards developing antivirals but holds great promise also for other diseases for which modulating RNA function has been shown to affect disease outcome.^17,18^

Rapid and large-scale (ScoV2) RNA genome sequencing has revolutionised the availability of the sequence data and thus contributes towards understanding the dynamics of viral evolution. Finding small molecules that can bind to conserved and structured regions of the non-coding parts of a viral RNA with high affinity and specificity is a powerful strategy to target the untranslated part of the genome of SCoV2, for which few mutations have been reported up to now.

The genome of SCoV2 contains four regulatory stem-loops in the 5′-untranslated region (5′-UTR), which are highly conserved among betacoronaviruses.^19–21^ Targeting such structured RNA elements to interfere with their regulatory function is regarded as a promising antiviral strategy, and targeting the SL1 stem-loop by antisense oligonucleotides has been shown to offer great therapeutic potential.^22–25^

In March 2020, the global consortium Covid19-NMR was formed.^26^ It was its mission to make viral proteins and RNAs amenable to nuclear magnetic resonance (NMR) spectroscopic studies including protein and RNA preparation and the optimisation of sample conditions. RNA secondary structures were determined for all conserved RNA elements along with their NMR-based resonance assignment, thus providing a basis for investigations of the interaction of small molecules or viral and host proteins with the viral genome.^27^ Two massive fragment screening campaigns involving 20 RNA elements and 25 proteins from SCoV2 resulted in the identification of 69 and 311 high-quality hits against the viral RNAs and proteins, respectively.^28–31^

**Fig. 1:**
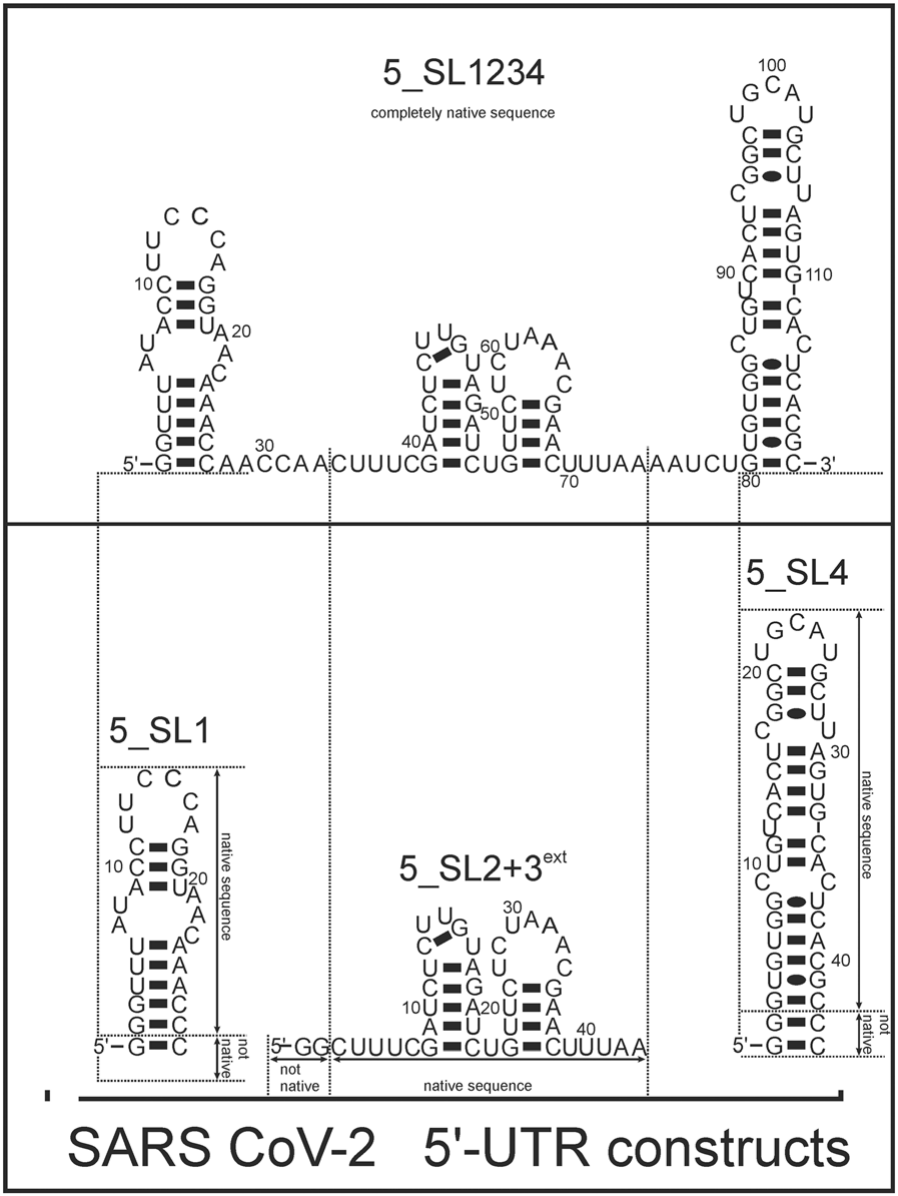
Schematic secondary structures of our chosen RNA sub constructs of the SCoV2 5′-UTR as previously determined.^27^

Here, we investigated and compared the general targetability of the 5′-terminal region of the 5′-UTR of SCoV2, consisting of the four stem-loops SL1, SL2, SL3, and SL4 (5_SL1234) and its three constituting sub-elements 5_SL1, 5_SL2 + 3^ext^, and 5_SL4 (Fig. 1) using an RNA-dedicated library^32^ (DRTL-F library) and a non-RNA-dedicated fragment library (DSI-PL).^33,34^ The DSI-PL and the DRTL-F library consist of 768 compound fragments (200–250 Da) and 49 compounds (222–439 Da), respectively.

In general, NMR spectroscopy is well suited for fragment screening and many such screens have been conducted in academia and in the pharmaceutical industry.^30,31,35^ The ability to detect weak binding, the comparably low amount of sample required and the ability to detect binding to non-modified molecular targets including proteins, nucleic acids and their complexes in a non-destructive manner in solution are particular advantages of NMR-based fragment screening. In addition to ^1^H-detected NMR experiments, ^19^F-detected NMR experiments have been recently advertised because binding can often be monitored on a single, background-free ^19^F signal in ^19^F-1D NMR experiments. ^19^F resonances show chemical shift dispersions that are large compared to the ^1^H or ^13^C chemical shift dispersions, allowing detection of binding-induced chemical shift perturbations (CSPs) and screening of mixtures containing a larger number of molecules compared to screening mixtures if ^1^H detection is used.^35,36^ Compared to ^13^C detection, ^19^F offers two advantages: ^19^F is a pure isotope (100% natural abundance) and it has a comparatively high sensitivity. The combined readout of ^1^H- & ^19^F-CSPs provides more reliable data for RNA screening in comparison to conventional STD experiments used for protein screening, since reduced proton density within RNA in contrast to protein leads to reduced STD effects upon (transient) binding.^37,38^

The goals of our study reported were:

(i) To compare the hit rates of the DSI-PL *versus* the DRTL-F library.

(ii) To investigate the validity of using isolated RNA sub-elements for such NMR-based screening approaches.

(iii) To evaluate the utility of ^19^F and ^1^H chemical shift perturbations (CSPs) and changes in *T*_2_ relaxation rates upon binding to RNA as proxies reporting on binding in NMR-based screening.

(iv) Evaluation of simultaneous NMR detection of CSPs for ^1^H and ^19^F ligands. Such dual detection scheme has become possible with the introduction of dual NMR receivers in the newest generation of NMR consoles. In principle, dual or parallel detection leads to a reduction of measurement time by a factor of two.^39^

We can show that the DRTL-F library is enriched in molecules that bind RNA and the binders derived from this library bind more tightly compared to molecules from the DSI-PL.

^19^F screening provides a small, but significant improvement in detecting binding compared to ^1^H screening, including detection and quantification of low affinity binding.

Most importantly, the RNA binders show selectivity for the 5_SL2 + 3^ext^ element of the 5′-terminal SCoV2 RNA. In order to map the RNA binding site to this preferred target RNA, we assigned the ^1^H,^15^N imino chemical shifts utilising NOESY, HSQC, and HNN-COSY experiments. These chemical shift assignments allow for rapid mapping of the RNA binding site by comparing chemical shift changes upon addition of RNA binders.

## Experimental

### RNA synthesis and purification

All RNA constructs of SCoV2 were synthesised by *in vitro* transcription using T7-polymerase and purified under the same conditions. First, DNA sequences for 5_SL1, 5_SL2 + 3^ext^, 5_SL4 and 5_SL1234 were obtained by cloning DNA oligomers encoding the T7 polymerase promotor (5′-TAATACGACTCACTATAG-3′) these RNA constructs into a plasmid based on the pSP64 vector. The plasmids also encode an HDV ribozyme, and the genes of interest were cloned into the plasmid using the EcoRI and NcoI restriction sites upstream of the ribozyme sequence to allow transcription of the RNA as an HDV fusion construct to avoid 3′ inhomogeneities of the RNA constructs.^40^

Transcriptions were performed at 15 mL scale, and the conditions were optimized for maximum yield and purity for each construct. Purification was carried out by preparative PAGE, HPLC and buffer exchange with screening buffer (25 mM KPi; 50 mM KCl; pH 6.2). After the PAGE, the desired RNA was visualized by UV-shadowing, cutted out and eluted with 0.3 M NaOAc. By using rpHPLC with a Kromasil RP 18 column and a gradient from non-polar to polar (0.1 M acetonitrile/triethylammonium acetate), the remaining PAA was removed. Concentrations of the samples were analysed by UV/vis spectroscopy using the following molar extinction coefficients (OD_260_): 5_SL1: 257.4 1/mM; 5_SL2 + 3^ext^: 390.0 1/mM; 5_SL4: 388.7 1/mM and 5_SL1234: 1064.1 1/mM, and analytical PAGE was performed to verify the purity.

### Sample preparation

For each RNA screened, there were two samples (one with and one without the fragment to be screened). Each screening sample contained 4 μM of RNA, 5% of D_2_O and 5% of [*d*_6_]DMSO, and the fragment at 0 μM or 40 μM concentration resulting in an [RNA]/[ligand]-ratio of 1 : 10. The screening buffer was 25 mM KPi (pH 6.2), 50 mM KCl in H_2_O. Titration samples contained 25 μM of the fragment, 5% [*d*_6_]DMSO (which was used as lock solvent), and RNA at concentrations from 0–125 μM.

### NMR spectroscopy

Spectra were recorded on a 600 MHz Bruker spectrometer equipped with a Neo console and a 5 mm cryogenic quadruple resonance ^1^H[^13^C,^15^N,^19^F] QCI probe and a SampleJet automatic sample changer. Measurements were performed in 3 mm sample tubes with a final volume of 170 μL. ^1^H 1Ds, ^19^F 1Ds and ^19^F *T*_2_-CPMG experiments were measured to identify binders (^19^F experiments were only measured for the fluorinated compounds). CPMG measurements were conducted with mixing times of 0, 16, 64, 128 and 256 ms.^35,41,42^ For the CSP evaluation, titrations were performed by ^1^H 1D measurements (and ^19^F 1D for all fluorinated fragments) on 8 samples with RNA concentrations varying from 0–125 μM and fixed ligand concentration of 25 μM. All NMR-measurements were conducted at 298 K. 5% [*d*_6_]DMSO was used as locking solvent for the spectrometer and shim optimization was done for every sample in an identical manner with a shimming-script loaded into the IconNMR-Automation Software. In ^1^H 1D screening experiments as well as titration experiments, a conventional excitation sculpting sequence was used to suppress the water.^43^^1^H 1D experiments were performed with 16k points, 1k scans, a spectral width of 8 kHz and a 53 min runtime each. ^19^F 1D experiments were performed with 150k points, 256 scans, a spectral width of 75 kHz and 9 min runtime. All data were processed and analysed using the software package TopSpin 4.0.7.

To assign the imino region of the 5_SL2 + 3^ext^ RNA, an ^1^H–^1^H-NOESY, an ^1^H–^15^N-BEST-TROSY and an HNN-COSY experiment were conducted. The spectra were recorded with 430 μM ^15^N-labelled 5_SL2 + 3^ext^ RNA. The experiments were performed at a 800 MHz Bruker spectrometer equipped with an Avance III HD console and a 5 mm cryogenic triple resonance ^1^H[^13^C, ^15^N] TCI probe. The measurements were carried out with the RNA in RNA buffer (25 mM KPi, pH 6.2 and 50 mM KCl) as much as 95% H_2_O/5% D_2_O. The ^1^H–^1^H-NOESY was run for 30 h with 400 points in the indirect dimension and 256 scans. The ^1^H–^15^N-BEST-TROSY was performed with 256 points in the indirect dimension and 16 scans for 35 min. The HNN-COSY was conducted with 192 points in the indirect dimension and 64 scans for 11 h runtime. All three spectra were recorded at 283 K.

For NMR-based binding site mapping detecting changes in the NMR spectra of G and U imino resonances in ^1^H,^15^N heteronuclear correlation experiments, two ^1^H–^15^N-BEST-TROSY spectra were recorded with 84 μM ^15^N-labelled 5_SL2 + 3^ext^ RNA alone and after addition of 420 μM ligand (ratio 1 : 5) under equal conditions. The measurements were run with a 800 MHz Bruker spectrometer equipped with an Avance III HD console and a 5 mm cryogenic triple resonance ^1^H[^13^C, ^15^N] TCI probe. The sample buffer was 25 mM KPi, pH 6.2 and 50 mM KCl. For locking of the spectrometer frequency, 5% D_2_O was added to the pure RNA sample and 5% D_2_O plus 5% [*d*_6_]DMSO was added to the RNA–ligand sample. Both measurements of the RNA alone and RNA plus ligand were performed with 256 points in the indirect dimension, 32 scans and 45 min runtime.

The dual detection experiments were performed on a 600 MHz spectrometer equipped with a Bruker Avance Neo console and a cryogenic probe for ^1^H, ^19^F [^13^C, ^15^N] (QCI). This type of console combines frequency generation with a digital receiver for each channel to ease implementation of dual receive experiments. Individual reference ^1^H and ^19^F-1D experiments were acquired with 512 scans and a relaxation delay of 1.4 s at an acquisition time of 1 s (^1^H) and 0.5 s (^19^F) in 20 min (^1^H) and 16 min (^19^F). The same settings were used for the dual receive experiment, recorded in 20 min. Water suppression for the proton experiment was achieved with a SOGGY sequence.^44^ This sequence was shown to be robust for use in screening experiments.^31^ The measurements were carried out with the ligand DRTL A04 at a concentration of 100° μM in RNA buffer and 95% H_2_O/5% DMSO.

### Analysis of NMR data

**Fig. 2:**
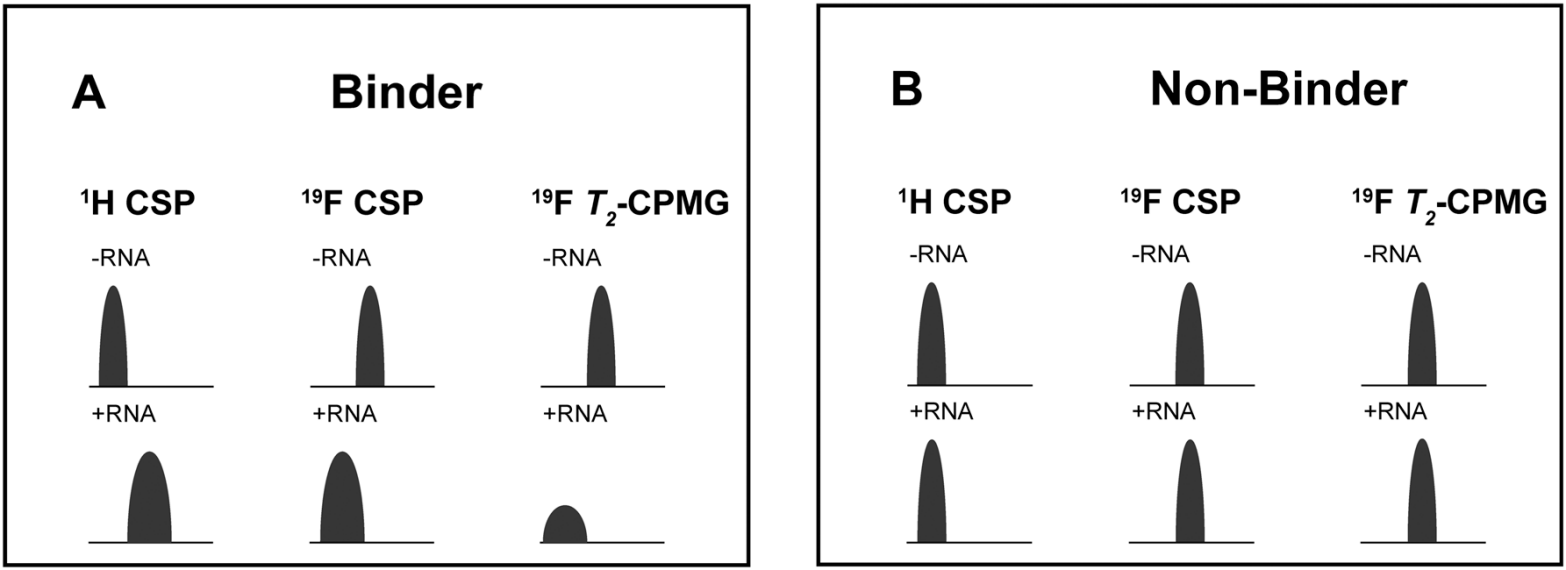
Ligand-based NMR screening and binding classification: schematic illustration of conducted experiments and criteria to identify binders A and non-binders B.

For every screening sample, the following three experiments were conducted: ^1^H 1D, ^19^F 1D and ^19^F *T*_2_-CMPG (Fig. 2). As hit criterion, we defined changes in all three parameters to be required (CSP (^1^H) ≥ 3 Hz; CSP (^19^F) ≥ 5 Hz; intensity decrease in the ^19^F *T*_2_-CPMG experiment ^19^F *T*_2_*Q*. ≥ 30%).^35,41,42^ The *T*_2_-reduction in percentage between peak integrals of samples containing ligand and RNA and sample containing only ligand with each 16 ms CPMG and 256 ms CPMG was calculated for all screening samples.
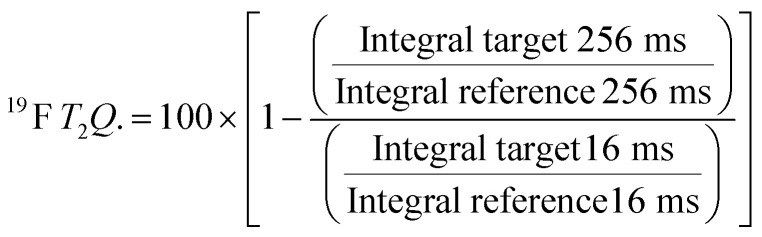
where “integral target” describes ligand with RNA and “integral reference” is only the ligand peak integral.

CSP-based calculation of the estimated dissociation constant (*K*^est^_D_) was done by plotting the changes in CSP values during NMR-titration experiments against RNA concentration and non-linear fitting. For this analysis, OriginLab Software was used.
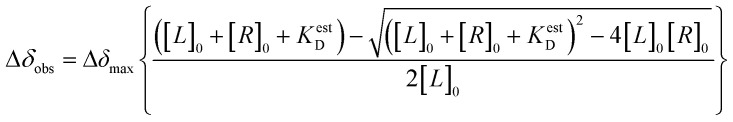
We report estimated *K*_D_ values because the concentration-dependent CSPs often do not reach saturation.

## Results

### Dual-detected NMR experiments

**Fig. 3:**
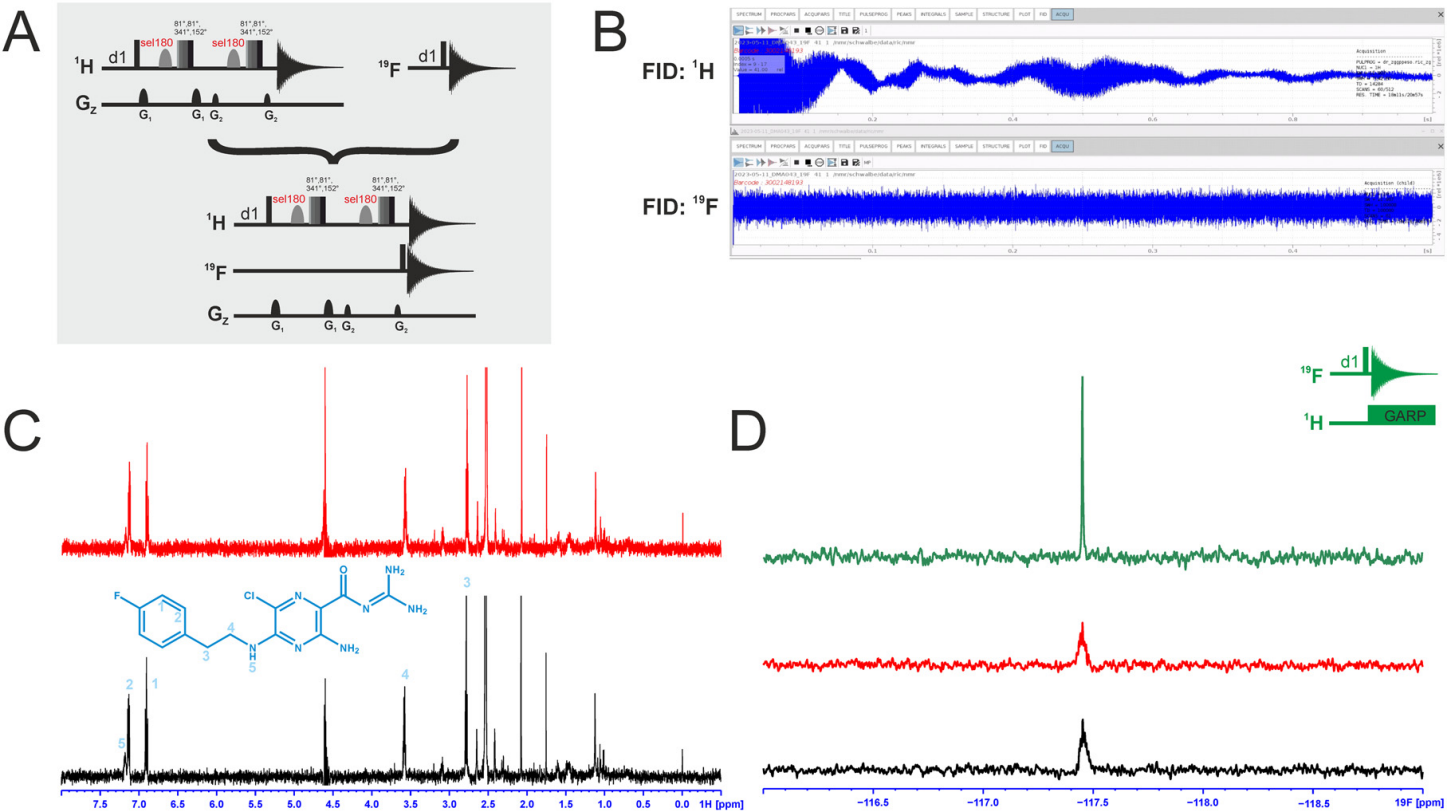
A: Pulse sequences for the single-receive experiments and the multi-receive experiment, respectively. B: FIDs of the multi-detected experiment with a shorter acquisition time for ^19^F. C: The individually recorded ^1^H-1D spectrum in red and the ^1^H-1D from the multi-detected experiment in black. Relevant signals of the compound are assigned. D: ^19^F-1D spectrum from the multi-detected experiment in black, the single recorded spectrum in red and the ^19^F-1D with ^1^H decoupling in green. The experiments were recorded with 512 scans at a field strength of 600 MHz.

We attempted to improve NMR screening technologies utilising dual detection schemes that allow detection of the ^1^H and the ^19^F at the same time (Fig. 3).^45^ In principle, such dual detection reduces the measurement time by combining detection of the response of two different NMR signals upon addition of ligands at the same time. Implementation of such approach, while feasible in principle, meets several challenges.

Typically, sensitivity of probes can only be optimised for detection of a single nucleus at a time. Thus, ^19^F-detected experiments require the inner coil to be tuned to ^19^F so that the detection coil picks up the rf-output of the ^19^F NMR signals, optimally exploiting the largest filling factor.

The presence of ^1^H–^19^F long-range couplings (^*n*^J(H,F) with *n* = 2–5) leads to splittings of both, ^1^H and ^19^F spectra that, while being information-rich, require expert analysis. Thus, typically ^1^H decoupling is used employing a second outer coil for probes that are optimised for ^19^F detection. Probes optimised for ^1^H detection can implement only band-selective ^1^H decoupling. If both nuclei are detected at the same time in dual detection mode, decoupling of either one of the two nuclei cannot be implemented. Decoupling is, however, not required for molecules that contain CF_3_-groups as these methyl groups typically do not feature large ^*n*^J(H,F) couplings.

We tested the feasibility of dual ^1^H and ^19^F detection. The DRTL-F (RNA-dedicated) library contains compounds with CF_3_-groups as well as fluorinated aromatic systems.

Simultaneous detection of ^1^H and ^19^F NMR signals was established using DRTL A04 as ligand (Fig. 3C). Each acquisition parameter was optimised individually and recorded under these conditions and compared with the parallel detected spectra (Fig. 3C and D). The signal-to-noise ratio (S/N) is identical in both cases for ^1^H and ^19^F. DRTL A04 carries a fluorine atom in a 1,4 di-substituted aromatic ring. ^2^J(H,F) and ^3^J(H,F) couplings cannot be suppressed in these experiments, so the S/N for the ^19^F experiments is worse compared to the decoupled ^19^F-1D. The less sensitive ^19^F-CPMG experiment can therefore not be performed in a reasonable time. Thus, while dual detection experiments can be performed for molecules carrying CF_3_ ligands, for ligands with sizeable scalar J(H,F) couplings, gains in measurement time due to dual detection cannot be realised as the heteronuclear couplings cannot be decoupled.

### Ligand-based screening *via* NMR spectroscopy

We screened two fragment libraries for binding to four RNA constructs derived from the 5′-UTR of the SCoV2 genome using ligand-based NMR experiments detecting either of the two NMR-active isotopes ^1^H or ^19^F.^19,27,36,46^ Before screening the various ligands, we verified that DMSO-induced shifts up to the volume percentage of 5% used in the screening experiments did not affect the chemical shifts of the RNA signals (ESI† Fig. S1).

The experimental design allows comparison of screening results based on either of the two detected NMR nuclei in parallel and in comparison to each other. Two libraries were used for screening: (i) the DSI-PL of fragments with 768 compounds, and (ii) the DRTL-F library developed by the Hargrove lab,^32^ containing 49 compounds.

While quality control NMR had already been conducted for the first library, we also conducted quality control NMR experiments for the DRTL-F library, in particular to determine solubilities of all compounds in the RNA NMR screening buffer by ^1^H 1D and ^19^F 1D spectroscopy.^34^

### Definition for binding within the screening

**Fig. 4:**
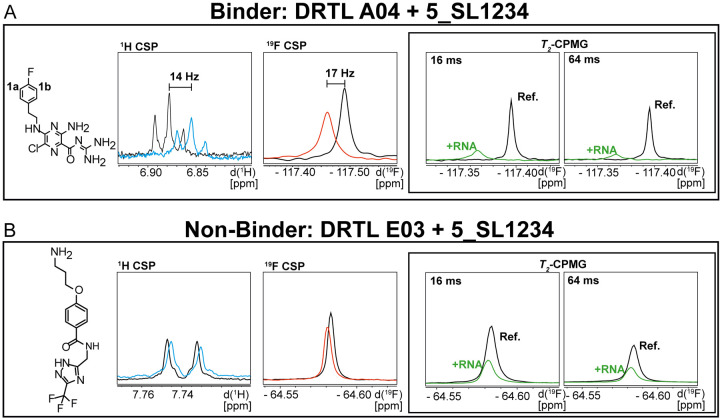
Chemical structures of DRTL A04 defined as binder (A) and DRTL E03 classified as non-binder (B) and their spectra (1D ^1^H, 1D ^19^F, *T*_2_-CPMG ^19^F after 16 ms and 64 ms) without (black) and in presence of 5_SL1234 RNA (coloured).

First, we screened the 4-stem-loop construct 5_SL1234 containing 119 nucleotides against both libraries. The most pronounced effects were observed for binding of compound DRTL A04 to 5_SL1234. We detected large CSPs in the ^1^H 1D (14 Hz) and the ^19^F 1D (17 Hz) and a signal decrease the ^19^F signal at ([ligand] : [RNA] = 10 : 1) for *T*_2_ relaxation times of 16 ms or 64 ms, respectively, compared to the reference signal without the RNA (Fig. 4A).

For comparison, we show the signature of the compound DRTL E03 that we classified as non-binder because neither a significant CSPs nor strong signal intensity attenuation in the *T*_2_-CPMG experiment could be detected (Fig. 4B).

**Fig. 5:**
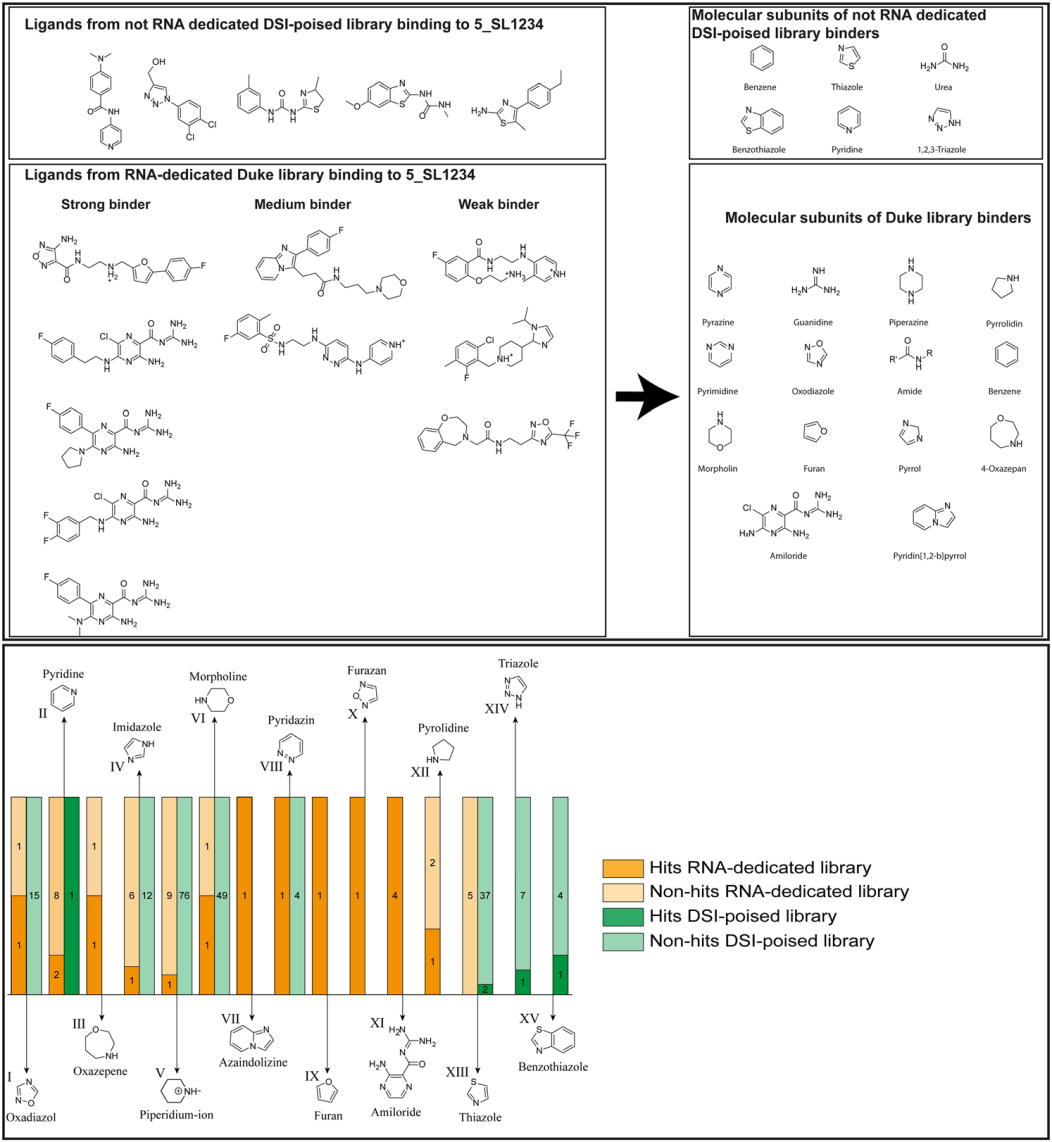
Comparison of all DRTL-F library and DSI-PL binders and their molecular subunits and chemotype comparison for the hits of both libraries.

Applying the hit criteria defined in Fig. 2A, we found 10 binders out of the 49 compounds from the DRTL-F library (Fig. 5). 5 out of 768 fragments of the DSI-PL were reported to bind previously.^30^ These numbers translate to a hit rate of 20.4% for the DRTL-F library *versus* 0.7% for the DSI-PL. The binding profiles of the 10 compounds from the DRTL-F library (Table S1†) and the 5 compounds from the DSI-PL (Table S2†) binding to 5_SL1234 are given in the ESI.†

In the next step, we screened the three sub-elements 5_SL1, 5_SL2 + 3^ext^ and 5_SL4 against the four most promising binders to 5_SL1234 from the DRTL-F library. All these ligands were amiloride derivatives.^47,48^

Apparently, those four amilorides did not bind to all three RNA sub-elements (Table 1), although they were found to be strong binders in the screening with the entire 5_SL1234 RNA before. This finding confirms that there is binding specificity for structured RNA motifs. The secondary structures of the three RNA sub-elements 5_SL1, 5_SL2 + 3^ext^ and 5_SL4 differ significantly: the secondary structure of 5_SL1 features an internal loop, 5_SL4 shows pyrimidine mismatches and 5_SL2 + 3^ext^ is the most flexible RNA sub-element and contains two structured stem regions and two loops of which one is a large loop.

**Table 1:** Results of screening the RNA sub-elements with the four most promising hits from the screening of 5_SL1234 with DRTL-F library. Binding can be derived from CSP values and the quotients of *T*_2_ reduction

RNAs	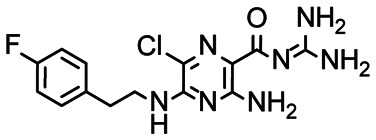			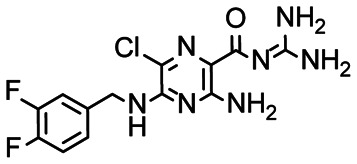			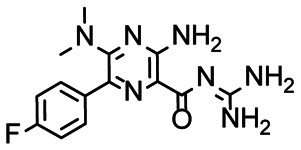			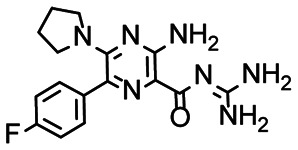		
	DRTL A04			DRTL B04			DRTL D04			DRTL A05		
	^1^H CSP	^19^F CSP	^19^F *T*_2_*Q*.	^1^H CSP	^19^F CSP	^19^F *T*_2_*Q*.	^1^H CSP	^19^F CSP	^19^F *T*_2_*Q*.	^1^H CSP	^19^F CSP	^19^F *T*_2_*Q*.
5_SL1234	14	17	78	13	27	96	26	6.5	100	20	1.6	100
5_SL1	3	10	0	0	11	78	1	2	2	2	7	20
5_SL2 + 3^ext^	8	24	100	3	17	100	0.7	0	39	1.8	7	43
5_SL4	3	13	17	3	7	92	2	2	48	0.8	6	11

Binding of amilorides to RNA involves electrostatic interactions. Our initial screening protocol uses a buffer system containing 50 mM KPi and 25 mM KCl, in accordance with optimal NMR spectral quality. We thus tested the influence of increased salt concentration up to 250 mM KCl on the binding of DRTL A04 to SL2 + 3^ext^. Interestingly, we observe largest ^1^H and ^19^F CSPs and substantially increased line widths at the lowest salt concentration. These large CSPs become smaller at higher salt concentration, but the large line widths remain. Such salt dependence on the primary NMR output could be consistent with more unspecific binding events involving more than a single site at higher KCl salt concentration (ESI† Fig. S2).

Interestingly, 5_SL2 + 3^ext^ binds two out of four screened compounds following the criteria applied for 5_SL1234, while 5_SL1 binds none and 5_SL4 only binds to DRTL B04 (Table 1).

While five fragments of the DSI-PL showed binding to 5_SL1234, there was no binder found at all in the screening of that library for 5_SL2 + 3, five for 5_SL1 and seven for 5_SL4.

Apparently, the (not-RNA dedicated) DSI-PL contains less compounds that bind RNA compared to the RNA-dedicated DRTL-F library. Closer analysis of the constitution of the binding compounds found in the two libraries shows that chemotypes for RNA binding differ as well (Fig. 5).

**Fig. 6:**
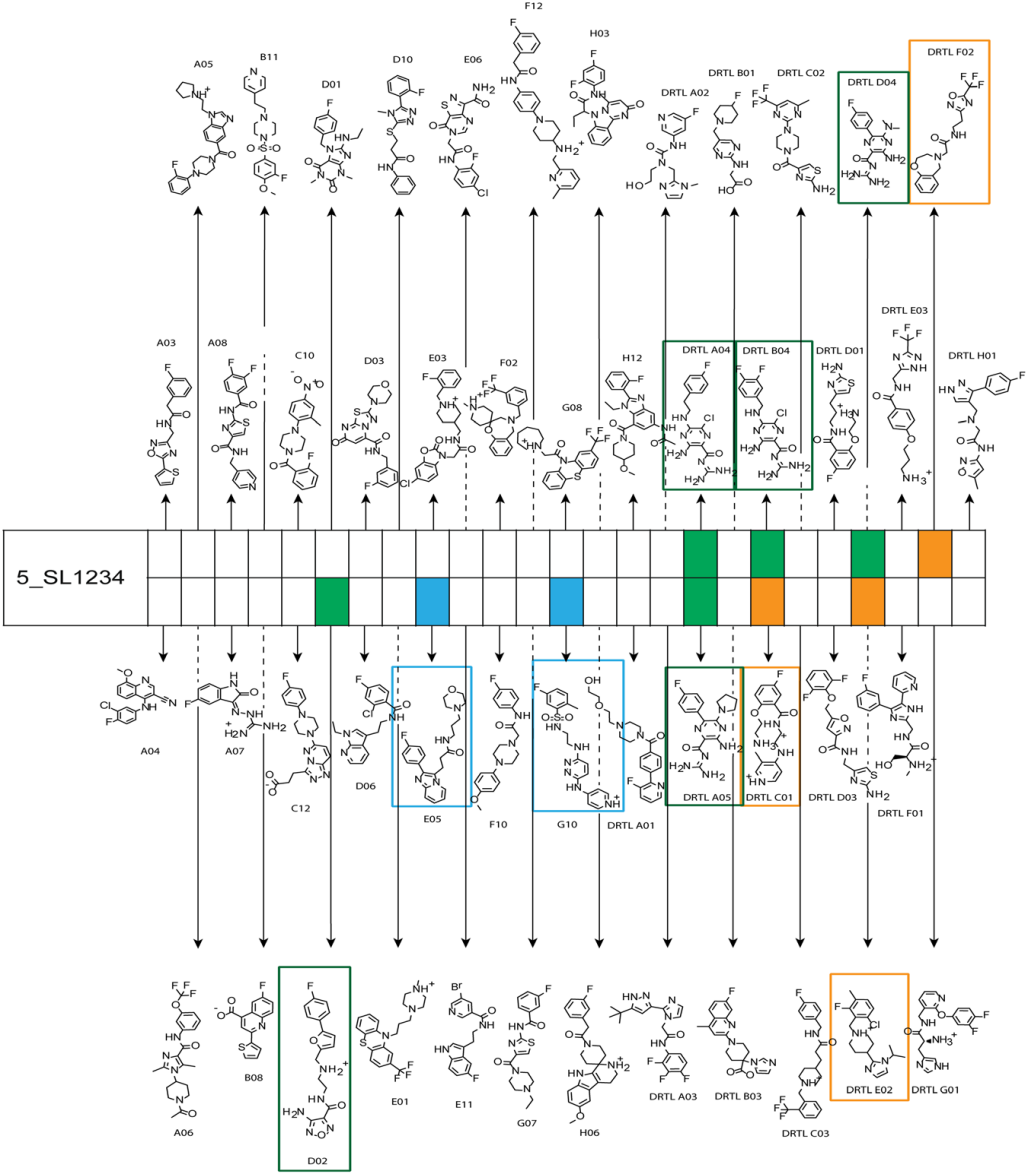
Overview of screening results from screening of 5_SL1234 with 49 compounds of the DRTL-F library. Strong binders are marked green, medium binders blue and weak binders yellow.

Fig. 5 summarises graphically the identified 15 chemotypes: Six chemotypes (III, VII, IX, X, XI) are unique for the DRTL-F library and two chemotypes (XIV, XV) are unique for the DSI-PL. The DSI-PL is enriched in several chemotypes (oxadiazol I, 15 fragments; imidazole IV, 12 fragments; piperidinium-ion V, 76 fragments; morpholine VI, 49 fragments) that are not found within its binders but are part of the binders of the DRTL-F library. Thiazole fragment XIII is found in 39 DSI_PL fragments of which two bind but is found to not be part of a binder in the DRTL-F library. Chemotype V, piperidinium-ion, is also found in ten DRTL-F library compounds but is only present in one binder, while one of the two DRTL-F library compounds containing chemotype morpholine VI is found to bind. The 6-membered nitrogen-carrying chemotypes V and VI often increase solubility, but apparently do not contribute to RNA binding. The amiloride chemotype 11 within the DRTL-F library stands out for its binding capability and represents a high-affinity non-specific tag for RNA binding, at least in part because the acyl guanidinium group is capable of forming hydrogen bonds on the Hoogsteen-edge in particular for a guanine nucleobase within the target RNA. An overview of the compounds screened within the DRTL-F library is given in Fig. 6.

### Estimation of relative binding affinities by ^1^H and ^19^F NMR titration

**Fig. 7:**
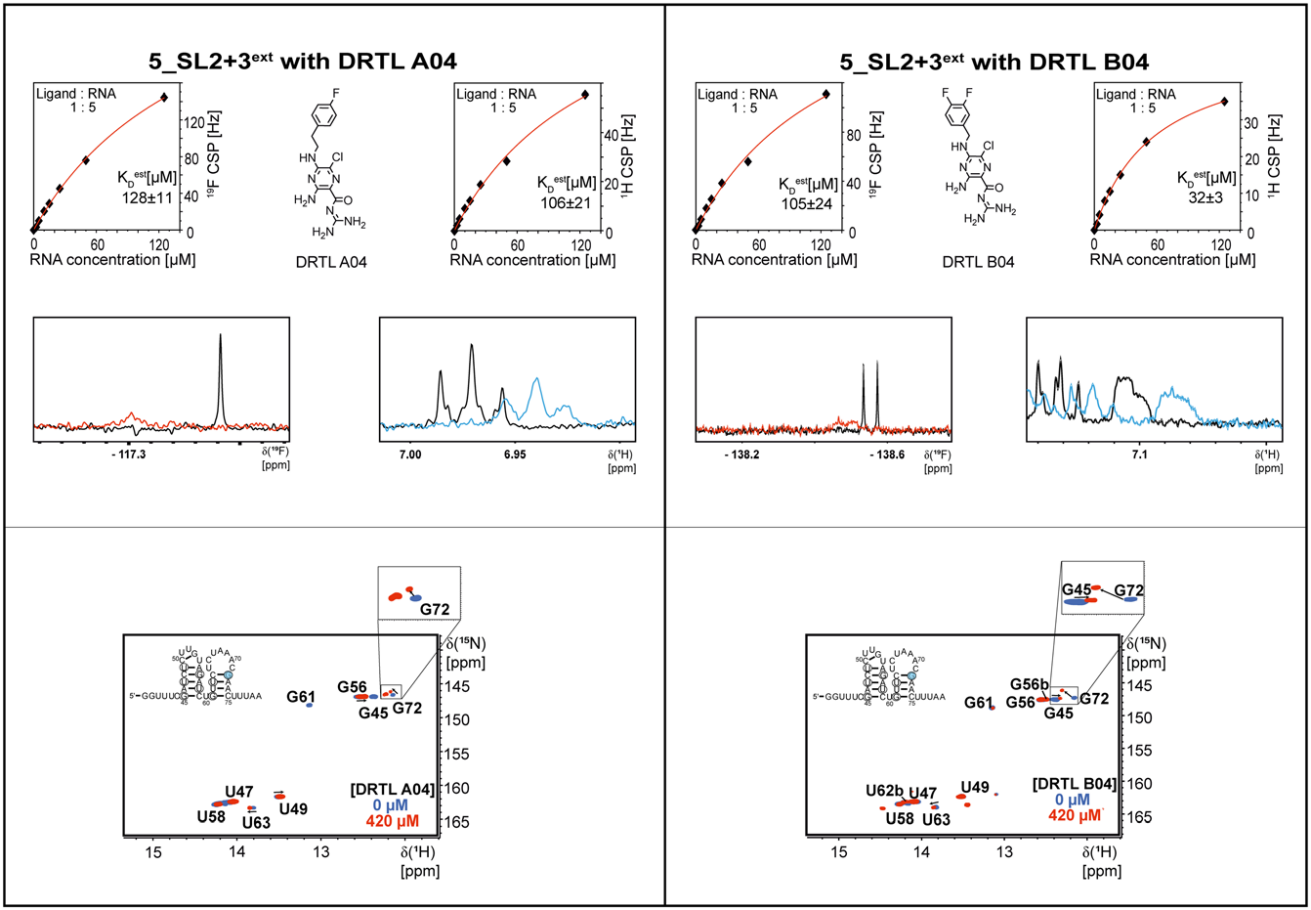
Fits of the chemical shift perturbations as function of RNA concentration from ^1^H and ^19^F 1D spectra of 5_SL2 + 3^ext^ and DRTL A04 and DRTL B04. 2D TROSY spectra overlays of RNA alone and RNA + ligand for 5_SL2 + 3^ext^ with DRTL A04 and DRTL B04.

The initial hit identification for the DRTL-F library was conducted using a tenfold excess of ligand over RNA target. We determined binding affinities for each of the amilorides prioritised for the RNAs to the three RNA sub-elements by ^1^H and ^19^F NMR titration. Here, the ligand concentration was fixed to 25 μM and increasing amounts of RNA were added up to a concentration of 125 μM. Compound CSPs were monitored for the titrations by ^1^H and ^19^F 1D spectra. Estimated *K*_D_ values (*K*^est^_D_) were calculated by fitting the CSPs as function of RNA concentration (Fig. 7). From analysis of the data, the sub-element 5_SL2 + 3^ext^ was found to be the best targetable RNA element. So, NMR-based binding site mapping was performed with 5_SL2 + 3^ext^ using changes of G and U imino resonances in ^1^H,^15^N heteronuclear correlation experiments of RNA alone compared to RNA with ligand as indicator of binding (Fig. 7). To do so, it was necessary to predominantly assign resonances of the imino region of 5_SL2 + 3^ext^, which was done by performing ^1^H–^1^H-NOESY, ^1^H–^15^N-BEST-TROSY and HNN-COSY experiments. Finally, since a small but detectable percentage of this RNA sub-element forms a secondary conformation, the experiments were performed at 283 K, which corresponds to the temperature range where the second conformation is least pronounced (ESI† Fig. S3).

To further compare the binding affinities of hits derived from either of the two libraries, the five fragments from the DSI-PL identified to bind to 5_SL1234 were titrated and affinities to the 5_SL2 + 3^ext^ were determined using ^1^H NMR of the non-fluorinated compounds. We found approximate affinities to range between 250 to 780 μM for these fragments (Table 2).

**Table 2:** Results of proton-detected NMR titration of 5_SL2 + 3^ext^ with the screening hits from the DSI-PL

RNA	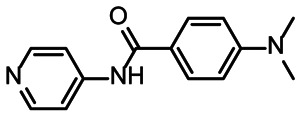	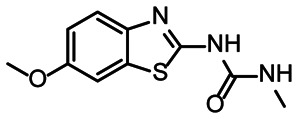	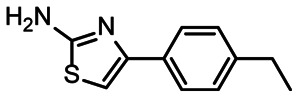	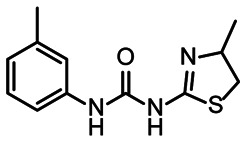	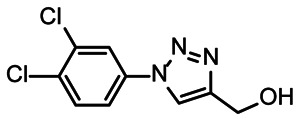
	Ligand 1	Ligand 2	Ligand 3	Ligand 4	Ligand 5
	^1^H estimated KD [μM]	^1^H estimated KD [μM]	^1^H estimated KD [μM]	^1^H estimated KD [μM]	^1^H estimated KD [μM]
5_SL2 + 3^ext^	255 ± 14	>1000	779 ± 365	>1000	>1000

NMR titration of hits identified in both libraries show that the dedicated RNA-library contains compounds that bind significantly stronger than those from the DSI-PL. Only for fragments ligand 1 and ligand 3 from the DSI-PL, binding affinities in the high micromolar range could be detected. By contrast, several low micromolar binding events for the amilorides of the DRTL-F library were observed.

**Fig. 8:**
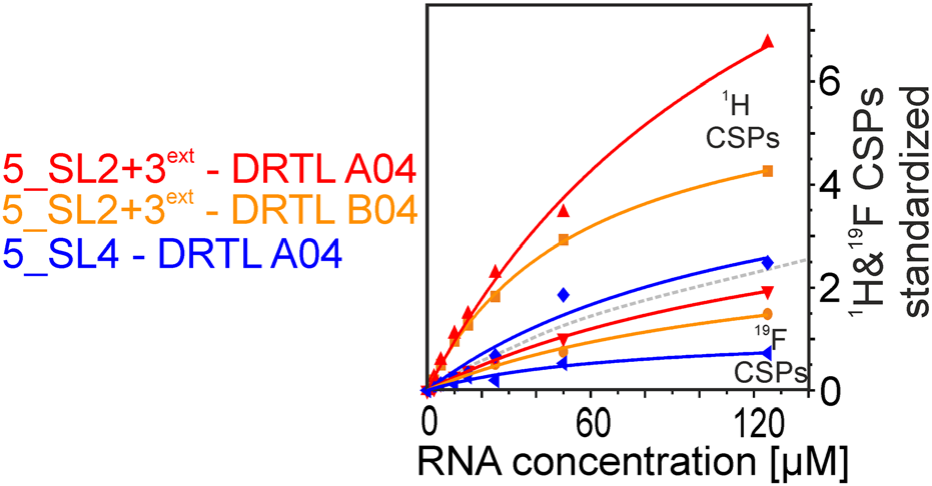
Estimated affinity plots of chemical shift perturbation against RNA concentration by stepwise titration of 5_SL2 + 3^ext^ and 5_SL4 to DRTL A04 and DRTL B04 *via*^1^H and ^19^F NMR over various RNA concentrations at a fixed compound concentration. CSP values were standardised with the observed spectral dispersion in Hertz of ^1^H or ^19^F 1D experiments, respectively. The grey dotted line indicates the border of the ranges of values between ^1^H and ^19^F CSPs induced by ligand binding.

We further compared the affinities determined by ^1^H *versus*^19^F CSPs for three RNA-compound pairs. The CSPs shown in Fig. 8 are standardised to the respective spectral dispersion
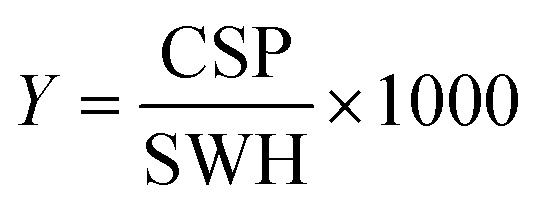
and show that the titration behavior for each of the nuclei ^1^H or ^19^F can be clustered and separated, indicated by the grey dotted line.

## Discussion and conclusion

**Fig. 9:**
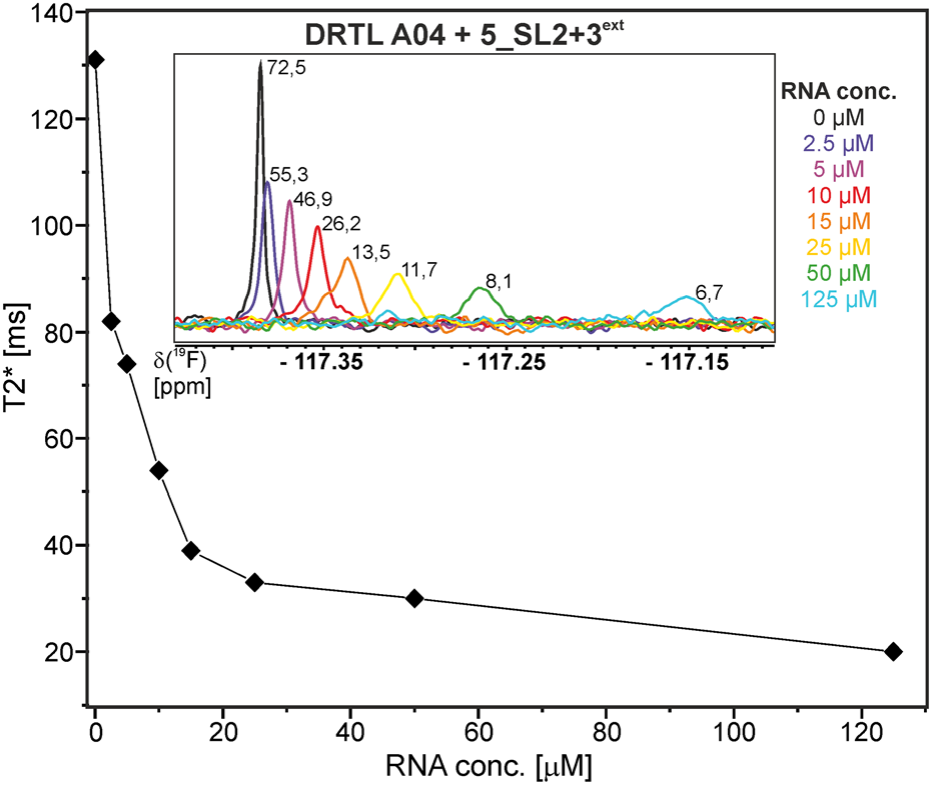
DRTL A04 ^19^F *T*_2_ relaxation times at a fixed ligand concentration of 25 μM and increasing 5_SL2 + 3^ext^ RNA concentrations from 0–125 μM. For [RNA] = 15 μM, the appearance of a shoulder peak is apparent. The signal-to-noise are indicated in the inlet showing the 1D spectra for each *T*_2_ mixing time.

Herein, we screened and titrated several compounds using ^1^H and ^19^F NMR to detect and characterise their binding to conserved RNA elements from the 5′UTR of the SCoV2 genome. All three elements of the 5_SL1234 untranslated region feature regular A-form secondary structures disrupted either by bulges or loops. Our findings suggest, that there must be binding specificity for either the structured RNA motifs of the sub-elements or unstructured spacer regions present in 5_SL1234 and not present in either of the sub-constructs. Each of the RNAs consists of unique structural elements. CSP can be detected both on the ^1^H and ^19^F resonances but weaker binding can be detected by ^19^F screening as its chemical shift dispersion (in Hertz) is larger than for ^1^H. Fig. 8 illustrates the CSP behaviour of ^1^H and ^19^F ligand resonances during titration with an RNA target. In addition, Fig. 9 shows the decrease in transverse relaxation *T*_2_ (^19^F) for DRTL A04 upon titration with 5_SL2 + 3^ext^. We found that standardisation of the observed CSPs with the spectral dispersion (accessible chemical shift range) for ^1^H and ^19^F, respectively, lead to a higher consistency between each compound–RNA-pair. In addition, after normalisation, the range of ^19^F CSPs is smaller and they are clustered.

Measuring both ^1^H and ^19^F experiments on identical samples allowed us to compare advantages and limitations with such experimental setup.

After detection of binding at one fixed concentration ratio of ligand over RNA ([ligand] : [RNA] = 10 : 1), binding affinities for those positive hits are characterised by suitable follow-up NMR experiments.

Here and in previous works, we propose to detect binding in a ligand-observed method by recording CSPs at a fixed ligand concentration and variable target concentrations.^28,30,31,34^ Especially for low affinity RNA binders (*K*_D_ of 100 μM–1 mM), estimates of ligand affinity are quick and easy to perform and provide a valuable checkpoint prior to performing detailed characterisation of the observed RNA binding. The latter method is more challenging in terms of sample amount, adjustment of experimental conditions, and ligand solubility. There is especially a problem with weaker binders that require a higher excess of RNA, where the ligand concentration must be sufficiently high to record NMR experiments within a reasonable time. Addition of DMSO to improve ligand solubility is limited: RNA folding, and thus binding site architecture, can be compromised at DMSO concentrations greater than 5%, which we often found to be the minimum required to keep compounds soluble during our experiments.

From the ligand-based affinity titrations conducted here, there are mainly two factors that determine the range of affinities that can be reliably quantified by NMR: signal-to-noise limits the lowest *K*_D_ that can be detected: from our experience, ^1^H and ^19^F experiments require ligands at a concentration of at least 10 μM. In ^1^H experiments, we prepared samples with [ligand] = 25 μM and added RNA up to a concentration of 125 μM.

Depending on the chemical shift of the ligand signal, we often observed spectral overlap in ^1^H spectra starting at a 1 : 1 ratio of compound to RNA. Obviously, this resonance overlap was circumvented in ^19^F-detected titrations. Additionally, ^19^F does not require solvent suppression and is not compromised by the presence of additional impurities from either RNA, buffer or ligand in general, although such impurities were not an issue in the work conducted here.

**Fig. 10:**
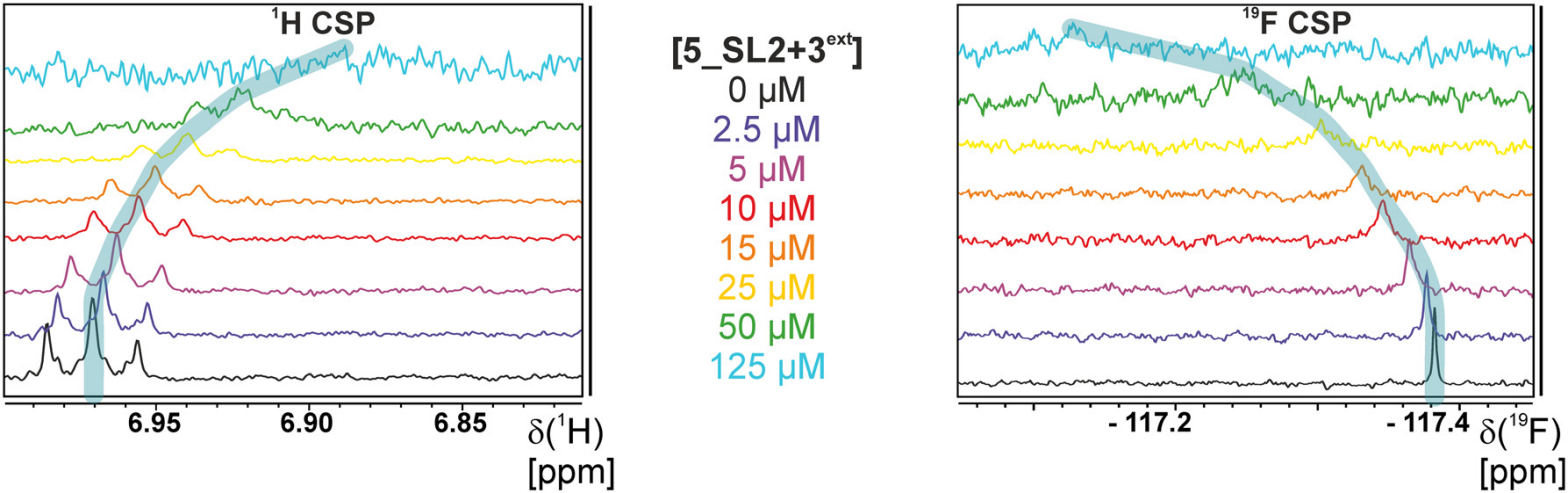
Overlayed 1D spectra of NMR titration ^1^H detected *versus*^19^F detected.

**Fig. 11:**
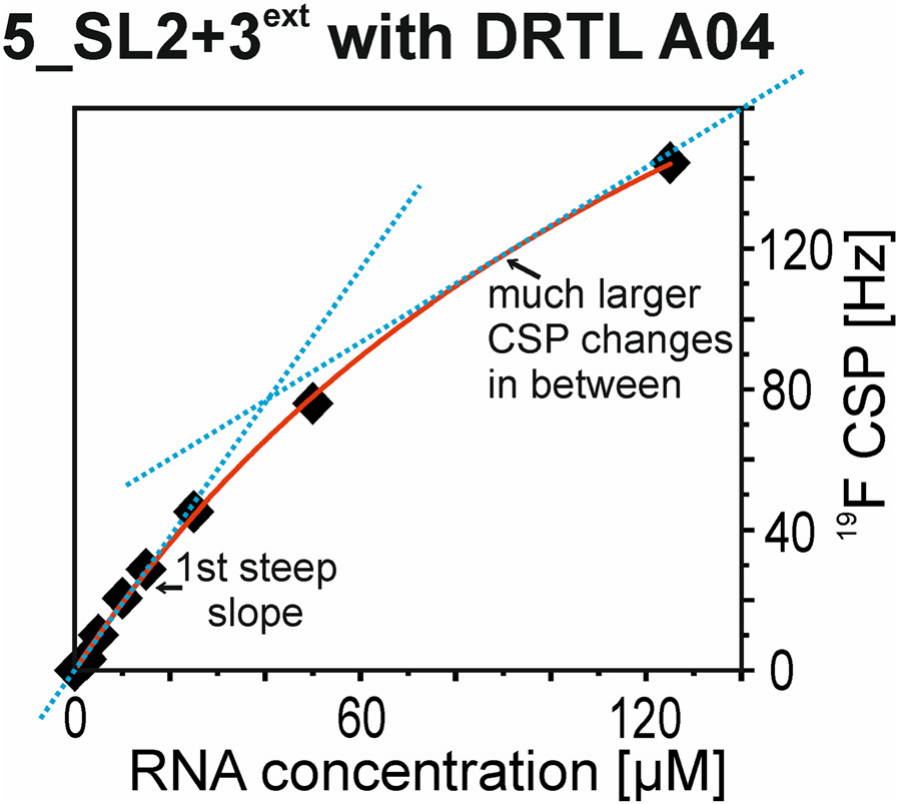
CSPs from ^19^F-detected NMR titration of 5_SL2 + 3^ext^ to DRTL A04 plotted against the RNA concentration. The blue dotted lines indicate two regions which differ in terms of CSP change in correlation to the RNA concentration.

The affinities we detect here represent relative estimates rather than highly precise *K*_D_*s*. Thus, we refer to estimated affinities (*K*^est^_D_), because the signal-to-noise ratio of the ligand signal often limits us with respect to the lowest possible ligand concentrations, which does not allow us to add sufficient excess of RNA to accurately determine the endpoints of the titration.^49^ Especially for ^19^F, we often observe substantial line broadening when RNA is added in excess over ligand (Fig. 10). This line broadening is presumably caused by several aspects: especially for ^19^F, the kinetics of the interaction change from fast exchange to intermediate exchange. The change in exchange regimes can be more readily observed for ^19^F than for ^1^H resonances, in line with larger chemical shift changes for ^19^F. In addition, some of the titration data suggest that the interaction of the ligand with RNA is not restricted to a single binding site, but two or several binding sites are involved. Here, we refrained from quantitatively describing these more complex interaction models, but especially for DRTL A04 binding to 5_SL2 + 3^ext^ (Fig. 11), we observed an initial steep CSP response followed by a second CSP change at higher RNA : ligand ratios.

From Fig. 11 it is apparent that there is a much larger region between the titration steps of 50 μM added and 125 μM added compared to the start of the curve where the titration steps, and respectively the gaps between CSP values, are much smaller. Thus, the chemical shift changes at higher RNA concentrations are larger than those at low RNA concentrations. This observation is consistent with a two-binding-site-scenario featuring a first binding site at ∼10 μM and a second, weaker binding and may account for frequent differences between NMR-detected affinities compared to other biophysical methods. In line with this, we observed different values for each RNA/fragment-pair depending on which of the CSPs we used (^1^H or ^19^F resonances) (Fig. 7 and 8). The reason for these differences could be that there are multiple binding events for the fragments to the RNA.

The exact nature of each individual interaction is likely different among all binding events, especially if the molecular encounter is driven by electrostatic or by stacking interactions, which are redundantly present in every RNA. The result is an ensemble of several local CSPs representing an average of all interaction events of either the fluorinated site of the compound or one proton resonance and RNA on the same timescale. In fact, we were able to detect such “delocalized” binding in our RNA-observed experiments (Fig. 7).

Towards the development of drugs targeting RNA optimised from initial fragment binders, we recommend using 1D and 2D NMR techniques to validate binding for those fragments that should be prioritised from initial ligand-based screens. NMR is particularly well suited for reliably selecting promising candidates in the lower affinity regime (3-digit μM) because it can distinguish between specific and non-specific binding.

The latter is a common issue with compounds targeting RNA rather than targeting proteins due to the large electrostatic contributions to binding and the inherent structural redundancy of many RNA motifs. Importantly, low-affinity binders that are selective for a particular RNA motif may be readily tuned to higher affinities for the same reasons, provided that detailed structural information about the binding site is available. Thus, by using the NMR methods presented here in the early stages of ligand development, the number of false positives leading to fruitless follow-up efforts can be reduced, and the same is true for the number of false negatives that may have been wrongly discarded. Especially when affinities in the single-digit micro molar range have been developed, additional biophysical methods need to be in place to accurately determine affinities. For example, fluorescent-based methods exploiting the inherent fluorescence of ligands or of fluorescent 2-aminopurine RNA residues introduced within the RNA target are options to pursue.

In conclusion, we demonstrate that RNAs can be targeted with substantial affinity and selectivity even by fragments. Enrichment of RNA binders in such fragment libraries accelerates the process of rapidly detecting binders in the 10–50 μM range. Our data support the notion that selectivity of 1–2 orders of magnitude in differential binding affinity can be reached over the set of RNAs investigated here even with low molecular weight molecules that are fragments but not yet optimized by extensive follow-up medicinal chemistry campaigns. The fluorinated library, that was used, is a subset of the Duke RNA-targeted Library (DRTL)^32^ with accompanying synthetic molecules and is not optimised for ease of NMR detection. Methodological NMR-centred screens often use compound libraries s containing only CF_3_-groups. The design of the library was guided in part by medicinal chemistry considerations including optimisation of affinity and selectivity as well as ease of follow-up chemistry in the hit-to-lead process and thus contain ^19^F groups where they may improve the binding properties of the hit but are not optimal for dual NMR detection. ^19^F screening provides advantages and limitations compared to ^1^H screening, we thus advocate to use both NMR screening technologies in parallel. While feasible in principle, dual detection schemes in NMR screening campaigns is hampered by insufficient S/N with the currently available probe technology and we recommend conducting individually optimised ^1^H and ^19^F experiments. However, methodological improvements are currently being developed to conduct such screens with a single sample strictly in parallel by dual detection methodology and with the right hardware and the correspondingly designed library, a lot of measurement time can be saved in the near future. The work reported will support the vast number of efforts to make also the RNA genome of viruses targetable by small molecules, both for mechanistic studies as an RNA target specific chemical probe but also towards antiviral medication. For such research, NMR spectroscopy is an indispensable tool to support medicinal chemistry campaigns.

## Author contributions

D. H., J. M., C. R., S. S., conducted research. N. N. P. synthesized the amiloride derivatives used. D. H., H. S. wrote the initial draft. All authors contributed in the iterative process of improving the initial draft. H. S. supervised the project. A. H. supervised synthesis and curation of the DRTL-F. A. H., H. S. acquired funding.

## Conflicts of interest

There are no conflicts to declare.

## Supplementary Material

MD-015-D3MD00322A-s001

## References

[cit1] Dong Y., Dai T., Wang B., Zhang L., Zeng L. H., Huang J., Yan H., Zhang L., Zhou F. (2021). Signal Transduction Targeted Ther..

[cit2] Painter G. R., Natchus M. G., Cohen O., Holman W., Painter W. P. (2021). Curr. Opin. Virol..

[cit3] Kozlov M. (2021). Nature.

[cit4] Liu J., Pan X., Zhang S., Li M., Ma K., Fan C., Lv Y., Guan X., Yang Y., Ye X., Deng X., Wang Y., Qin L., Xia Z., Ge Z., Zhou Q., Zhang X., Ling Y., Qi T., Wen Z., Huang S., Zhang L., Wang T., Liu Y., Huang Y., Li W., Du H., Chen Y., Xu Y., Zhao Q., Zhao R., Annane D., Qu J., Chen D. (2023). Lancet Reg. Health West. Pac..

[cit5] Kozlov M. (2022). Nature.

[cit6] Yu R., Chen L., Lan R., Shen R., Li P. (2020). Int. J. Antimicrob. Agents.

[cit7] Günther S., Reinke P. Y. A., Fernández-Garciá Y., Lieske J., Lane T. J., Ginn H. M., Koua F. H. M., Ehrt C., Ewert W., Oberthuer D., Yefanov O., Meier S., Lorenzen K., Krichel B., Kopicki J. D., Gelisio L., Brehm W., Dunkel I., Seychell B., Gieseler H., Norton-Baker B., Escudero-Pérez B., Domaracky M., Saouane S., Tolstikova A., White T. A., Hänle A., Groessler M., Fleckenstein H., Trost F., Galchenkova M., Gevorkov Y., Li C., Awel S., Peck A., Barthelmess M., Schlünzen F., Xavier P. L., Werner N., Andaleeb H., Ullah N., Falke S., Srinivasan V., Francą B. A., Schwinzer M., Brognaro H., Rogers C., Melo D., Zaitseva-Doyle J. J., Knoska J., Penã-Murillo G. E., Mashhour A. R., Hennicke V., Fischer P., Hakanpaä J., Meyer J., Gribbon P., Ellinger B., Kuzikov M., Wolf M., Beccari A. R., Bourenkov G., Von Stetten D., Pompidor G., Bento I., Panneerselvam S., Karpics I., Schneider T. R., Garcia-Alai M. M., Niebling S., Günther C., Schmidt C., Schubert R., Han H., Boger J., Monteiro D. C. F., Zhang L., Sun X., Pletzer-Zelgert J., Wollenhaupt J., Feiler C. G., Weiss M. S., Schulz E. C., Mehrabi P., Karnïcar K., Usenik A., Loboda J., Tidow H., Chari A., Hilgenfeld R., Uetrech C., Cox R., Zaliani A., Beck T., Rarey M., Günther S., Turk D., Hinrichs W., Chapman H. N., Pearson A. R., Betzel C., Meents A. (2021). Science.

[cit8] Guven O., Gul M., Ayan E., Johnson J. A., Cakilkaya B., Usta G., Ertem F. B., Tokay N., Yuksel B., Gocenler O., Buyukdag C., Botha S., Ketawala G., Su Z., Hayes B., Poitevin F., Batyuk A., Yoon C. H., Kupitz C., Durdagi S., Sierra R. G., Demirci H. (2021). Crystals.

[cit9] Douangamath A., Fearon D., Gehrtz P., Krojer T., Lukacik P., Owen C. D., Resnick E., Strain-Damerell C., Aimon A., Ábrányi-Balogh P., Brandão-Neto J., Carbery A., Davison G., Dias A., Downes T. D., Dunnett L., Fairhead M., Firth J. D., Jones S. P., Keeley A., Keserü G. M., Klein H. F., Martin M. P., Noble M. E. M., O'Brien P., Powell A., Reddi R. N., Skyner R., Snee M., Waring M. J., Wild C., London N., von Delft F., Walsh M. A. (2020). Nat. Commun..

[cit10] Newman J. A., Douangamath A., Yadzani S., Yosaatmadja Y., Aimon A., Brandão-Neto J., Dunnett L., Gorrie-stone T., Skyner R., Fearon D., Schapira M., von Delft F., Gileadi O. (2021). Nat. Commun..

[cit11] Ursu A., Childs-Disney J. L., Andrews R. J., O'Leary C. A., Meyer S. M., Angelbello A. J., Moss W. N., Disney M. D. (2020). Chem. Soc. Rev..

[cit12] Maucort C., Vo D. D., Aouad S., Charrat C., Azoulay S., Di Giorgio A., Duca M. (2021). ACS Med. Chem. Lett..

[cit13] Kelly M. L., Chu C. C., Shi H., Ganser L. R., Bogerd H. P., Huynh K., Hou Y., Cullen B. R., Al-Hashimi H. M. (2021). RNA.

[cit14] Meyer S. M., Williams C. C., Akahori Y., Tanaka T., Aikawa H., Tong Y., Childs-Disney J. L., Disney M. D. (2020). Chem. Soc. Rev..

[cit15] Childs-Disney J. L., Yang X., Gibaut Q. M. R., Tong Y., Batey R. T., Disney M. D. (2022). Nat. Rev. Drug Discovery.

[cit16] Warner K. D., Hajdin C. E., Weeks K. M. (2018). Nat. Rev. Drug Discovery.

[cit17] Abudayyeh O. O., Gootenberg J. S., Essletzbichler P., Han S., Joung J., Belanto J. J., Verdine V., Cox D. B. T., Kellner M. J., Regev A., Lander E. S., Voytas D. F., Ting A. Y., Zhang F. (2017). Nature.

[cit18] Bennett C. F., Swayze E. E. (2010). Annu. Rev. Pharmacol. Toxicol..

[cit19] Rangan R., Zheludev I. N., Hagey R. J., Pham E. A., Wayment-Steele H. K., Glenn J. S., Das R. (2020). RNA.

[cit20] Yang D., Leibowitz J. L. (2015). Virus Res..

[cit21] Andrews R. J., O'leary C. A., Tompkins V. S., Peterson J. M., Haniff H. S., Williams C., Disney M. D., Moss W. N. (2021). NAR: Genomics Bioinf..

[cit22] Haniff H. S., Tong Y., Liu X., Chen J. L., Suresh B. M., Andrews R. J., Peterson J. M., O'Leary C. A., Benhamou R. I., Moss W. N., Disney M. D. (2020). ACS Cent. Sci..

[cit23] Hermann T. (2016). Wiley Interdiscip. Rev.: RNA.

[cit24] Richter C., Hohmann K. F., Toews S., Mathieu D., Altincekic N., Bains J. K., Binas O., Ceylan B., Duchardt-Ferner E., Ferner J., Fürtig B., Grün J. T., Hengesbach M., Hymon D., Jonker H. R. A., Knezic B., Korn S. M., Landgraf T., Löhr F., Peter S. A., Pyper D. J., Qureshi N. S., Schlundt A., Schnieders R., Stirnal E., Sudakov A., Vögele J., Weigand J. E., Wirmer-Bartoschek J., Witt K., Wöhnert J., Schwalbe H., Wacker A. (2021). Biomol. NMR Assignments.

[cit25] Joshi S., Parkar J., Ansari A., Vora A., Talwar D., Tiwaskar M., Patil S., Barkate H. (2021). Int. J. Infect. Dis..

[cit26] Duchardt-Ferner E., Ferner J., Fürtig B., Hengesbach M., Richter C., Schlundt A., Sreeramulu S., Wacker A., Weigand J. E., Wirmer-Bartoschek J., Schwalbe H. (2023). Angew. Chem., Int. Ed..

[cit27] Wacker A., Weigand J. E., Akabayov S. R., Altincekic N., Bains J. K., Banijamali E., Binas O., Castillo-Martinez J., Cetiner E., Ceylan B., Chiu L. Y., Davila-Calderon J., Dhamotharan K., Duchardt-Ferner E., Ferner J., Frydman L., Fürtig B., Gallego J., Grün J. T., Hacker C., Haddad C., Hähnke M., Hengesbach M., Hiller F., Hohmann K. F., Hymon D., de Jesus V., Jonker H., Keller H., Knezic B., Landgraf T., Löhr F., Luo L., Mertinkus K. R., Muhs C., Novakovic M., Oxenfarth A., Palomino-Schätzlein M., Petzold K., Peter S. A., Pyper D. J., Qureshi N. S., Riad M., Richter C., Saxena K., Schamber T., Scherf T., Schlagnitweit J., Schlundt A., Schnieders R., Schwalbe H., Simba-Lahuasi A., Sreeramulu S., Stirnal E., Sudakov A., Tants J. N., Tolbert B. S., Vögele J., Weiß L., Wirmer-Bartoschek J., Wirtz Martin M. A., Wöhnert J., Zetzsche H. (2020). Nucleic Acids Res..

[cit28] Berg H., Martin M. A. W., Altincekic N., Alshamleh I., Bains J. K., Blechar J., Ceylan B., de Jesus V., Dhamotharan K., Fuks C., Gande S. L., Hargittay B., Hohmann K. F., Hutchison M. T., Korn S. M., Krishnathas R., Kutz F., Linhard V., Matzel T., Meiser N., Niesteruk A., Pyper D. J., Schulte L., Trucks S., Azzaoui K., Blommers M. J. J., Gadiya Y., Karki R., Zaliani A., Gribbon P., da Silva Almeida M., Anobom C. D., Bula A. L., Bütikofer M., Caruso Í. P., Felli I. C., Da Poian A. T., de Amorim G. C., Fourkiotis N. K., Gallo A., Ghosh D., Gomes-Neto F., Gorbatyuk O., Hao B., Kurauskas V., Lecoq L., Li Y., Mebus-Antunes N. C., Mompeán M., Neves-Martins T. C., Ninot-Pedrosa M., Pinheiro A. S., Pontoriero L., Pustovalova Y., Riek R., Robertson A. J., Saad M. J. A., Treviño M., Tsika A. C., Almeida F. C. L., Bax A., Henzler-Wildman K., Hoch J. C., Jaudzems K., Laurents D. V., Orts J., Pierattelli R., Spyroulias G. A., Duchardt-Ferner E., Ferner J., Fürtig B., Hengesbach M., Löhr F., Qureshi N., Richter C., Saxena K., Schlundt A., Sreeramulu S., Wacker A., Weigand J. E., Wirmer-Bartoschek J., Wöhnert J., Schwalbe H. (2022). Angew. Chem., Int. Ed..

[cit29] Zafferani M., Haddad C., Luo L., Davila-Calderon J., Chiu L. Y., Mugisha C. S., Monaghan A. G., Kennedy A. A., Yesselman J. D., Gifford R. J., Tai A. W., Kutluay S. B., Li M. L., Brewer G., Tolbert B. S., Hargrove A. E. (2021). Sci. Adv..

[cit30] Sreeramulu S., Richter C., Berg H., Martin M. A. W., Ceylan B., Matzel T., Adam J., Altincekic N., Azzaoui K., Bains J. K., Blommers M. J. J., Ferner J., Fürtig B., Göbel M., Grün J. T., Hengesbach M., Hohmann K. F., Hymon D., Knezic B., Martins J. N., Mertinkus K. R., Niesteruk A., Peter S. A., Pyper D. J., Qureshi N. S., Scheffer U., Schlundt A., Schnieders R., Stirnal E., Sudakov A., Tröster A., Vögele J., Wacker A., Weigand J. E., Wirmer-Bartoschek J., Wöhnert J., Schwalbe H. (2021). Angew. Chem..

[cit31] Berg H., Wirtz Martin M. A., Niesteruk A., Richter C., Sreeramulu S., Schwalbe H. (2021). J. Visualized Exp..

[cit32] Wicks S. L. , Morgan B. S., Wilson A. W. and Hargrove A. E., Probing Bioactive Chemical Space to Discover RNA-Targeted Small Molecules, bioRxiv, 2023, preprint, 10.1101/2023.07.31.551350

[cit33] Vögele J., Ferner J. P., Altincekic N., Bains J. K., Ceylan B., Fürtig B., Grün J. T., Hengesbach M., Hohmann K. F., Hymon D., Knezic B., Löhr F., Peter S. A., Pyper D., Qureshi N. S., Richter C., Schlundt A., Schwalbe H., Stirnal E., Sudakov A., Wacker A., Weigand J. E., Wirmer-Bartoschek J., Wöhnert J., Duchardt-Ferner E. (2021). Biomol. NMR Assignments.

[cit34] Sreeramulu S., Richter C., Kuehn T., Azzaoui K., Blommers M. J. J., Del Conte R., Fragai M., Trieloff N., Schmieder P., Nazaré M., Specker E., Ivanov V., Oschkinat H., Banci L., Schwalbe H. (2020). J. Biomol. NMR.

[cit35] Binas O., de Jesus V., Landgraf T., Völklein A. E., Martins J., Hymon D., Kaur Bains J., Berg H., Biedenbänder T., Fürtig B., Gande S. L., Niesteruk A., Oxenfarth A., Qureshi N. S., Schamber T., Schnieders R., Tröster A., Wacker A., Wirmer-Bartoschek J., Martin M. A. W., Stirnal E., Azzaoui K., Richter C., Sreeramulu S., Blommers M. J. J., Schwalbe H. (2021). ChemBioChem.

[cit36] Dalvit C., Fagerness P. E., Hadden D. T. A., Sarver R. W., Stockman B. J. (2003). J. Am. Chem. Soc..

[cit37] Mayer M., Meyer B. (1999). Angew. Chem., Int. Ed..

[cit38] Mayer M., James T. (2002). J. Am. Chem. Soc..

[cit39] Kupče E., Freeman R., John B. K. (2006). J. Am. Chem. Soc..

[cit40] Schürer H., Lang K., Schuster J., Mörl M. (2002). Nucleic Acids Res..

[cit41] Carr H. Y., Purcell E. M. (1954). Phys. Rev..

[cit42] Meiboom S., Gill D. (1958). Rev. Sci. Instrum..

[cit43] Hwang T., Shaka A. (1995). J. Magn. Reson..

[cit44] Nguyen B. D., Meng X., Donovan K. J., Shaka A. J. (2007). J. Magn. Reson..

[cit45] Kovacs H., Kupče Ē. (2016). Magn. Reson. Chem..

[cit46] Pellecchia M., Bertini I., Cowburn D., Dalvit C., Giralt E., Jahnke W., James T. L., Homans S. W., Kessler H., Luchinat C., Meyer B., Oschkinat H., Peng J., Schwalbe H., Siegal G. (2008). Nat. Rev. Drug Discovery.

[cit47] Patwardhan N. N., Ganser L. R., Kapral G. J., Eubanks C. S., Lee J., Sathyamoorthy B., Al-Hashimi H. M., Hargrove A. E. (2017). MedChemComm.

[cit48] Patwardhan N. N., Cai Z., Umuhire Juru A., Hargrove A. E. (2019). Org. Biomol. Chem..

[cit49] Jarmoskaite I., Alsadhan I., Vaidyanathan P. P., Herschlag D. (2020). eLife.

