# Supplementary material for: NMR 1H,19F-based screening of the four stem-looped structure 5_SL1–SL4 located in the 5′-untranslated region of SARS-CoV 2 RNA
Source: RSC Med Chem. 2023 Nov 28;15(1):165–77. doi: 10.1039/d3md00322a (PMC10809358; doi:10.1039/d3md00322a)
Supplement: MD-015-D3MD00322A-s001 [file MD-015-D3MD00322A-s001.pdf]

Electronic Supplementary Material (ESI) for RSC Medicinal Chemistry.
This journal is © The Royal Society of Chemistry 2023

Electronic Supplementary Material (ESI) for RSC Medicinal Chemistry.

NMR ^{1}H,^{19}F-based screening of the four stem-looped structure 5_SL1-SL4 located in the 5'-untranslated region of SARS-CoV 2 RNA

Daniel Hymon[1], Jason Martins[1], Christian Richter[1], Sridhar Sreeramulu[1], Anna Wacker[1], Jan Ferner[1], Neeraj N. Patwardhan[2], Amanda E. Hargrove[2], Harald Schwalbe[1,*]

[1]Institute for Organic Chemistry and Chemical Biology, Center for Biomolecular Magnetic Resonance (BMRZ), Goethe-University Frankfurt, Max-von-Laue-Str. 7, 60438 Frankfurt/Main, Germany.

[2]Department of Chemistry, Duke University, Durham, North Carolina 27708, USA

Table 1 SI: CSP values and T_2 reduction quotients of all RNA binders from the DRTL-F library.

	compound	5_SL1234			5_SL1			5_SL2+3^{ext}			5_SL4		
		^{1}H CSP	^{19}F CSP	^{19}F T_2 Q.	^{1}H CSP	^{19}F CSP	^{19}F T_2 Q.	^{1}H CSP	^{19}F CSP	^{19}F T_2 Q.	^{1}H CSP	^{19}F CSP	^{19}F T_2 Q.
strong binders to 5_SL1234	DRTL A04	14	17	78	2.6	10	0	8	24	100	3	13	17
	DRTL B04	13	27	96	0	11	78	2.7	17	100	3	7	92
	DRTL D04	26	6.5	100	1	2	2	0.7	0	39	2	2	48
	DRTL A05	20	1.6	100	2	7	20	1.8	7	43	0.8	6	11
	D02	12	11.5	100									
medium binders to 5_SL1234	E05	5	18	100									
	G10	3.6	0.4	40									
weak binders to 5_SL1234	DRTL C01	14	1.5	59									
	DRTL E02	5	4.2	100									
	DRTL F02	0	8	2									

Table 2 SI: CSP, LOGSY factor andT2 reduction quotients of the 5_SL1234 binders from the DSI library

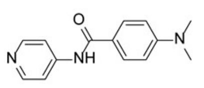
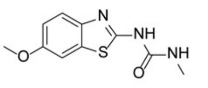
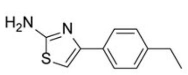
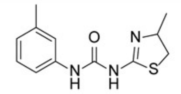
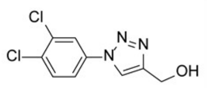

	Ligand 1			Ligand 2			Ligand 3			Ligand 4			Ligand 5		
	^{1}H CSP	LOGSY factor	^{1}H T_2 Q.	^{1}H CSP	LOGSY factor	^{1}H T_2 Q.	^{1}H CSP	LOGSY factor	^{1}H T_2 Q.	^{1}H CSP	LOGSY factor	^{1}H T_2 Q.	^{1}H CSP	LOGSY factor	^{1}H T_2 Q.
**5_Sl2+3^{ext}**	12.0	2.0	15%	8.0	1.1	68%	3.0	6.6	31%	11.0	6.2	91%	7.0	5.1	35%

Binding critieria: ^{1}H CSP ≥ 6 Hz; LOGSY factor > 1; ^{1}H T_2 Q. ≥ 20%. Hit identification when 2 of 3 criteria are fullfilled.

Table 3 SI: K_D^{est} of the three RNA subconstructs with with the four most promising binder from DRTL-F library.

		5_SL1	5_SL2+3^{ext}	5_SL4
DRTL A04	^{1}H K_D^{est} [µM]	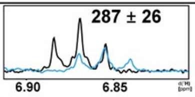	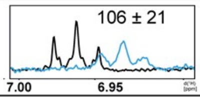	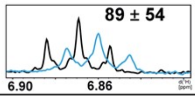
	^{19}F K_D^{est} [µM]	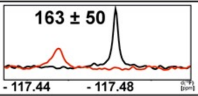	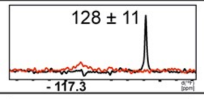	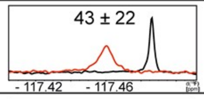
DRTL B04	^{1}H K_D^{est} [µM]		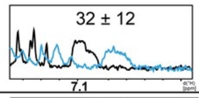	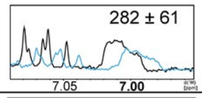
	^{19}F K_D^{est} [µM]		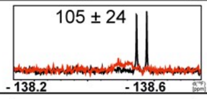	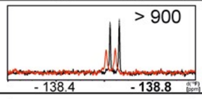
DRTL D04	^{1}H K_D^{est} [µM]		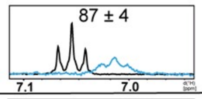	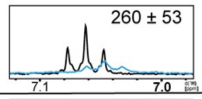
	^{19}F K_D^{est} [µM]		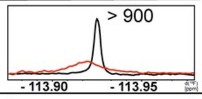	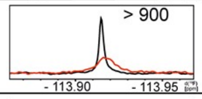
DRTL A05	^{1}H K_D^{est} [µM]		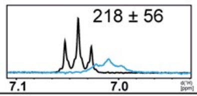	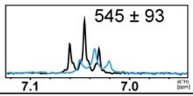
	^{19}F K_D^{est} [µM]		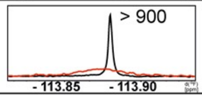	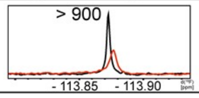

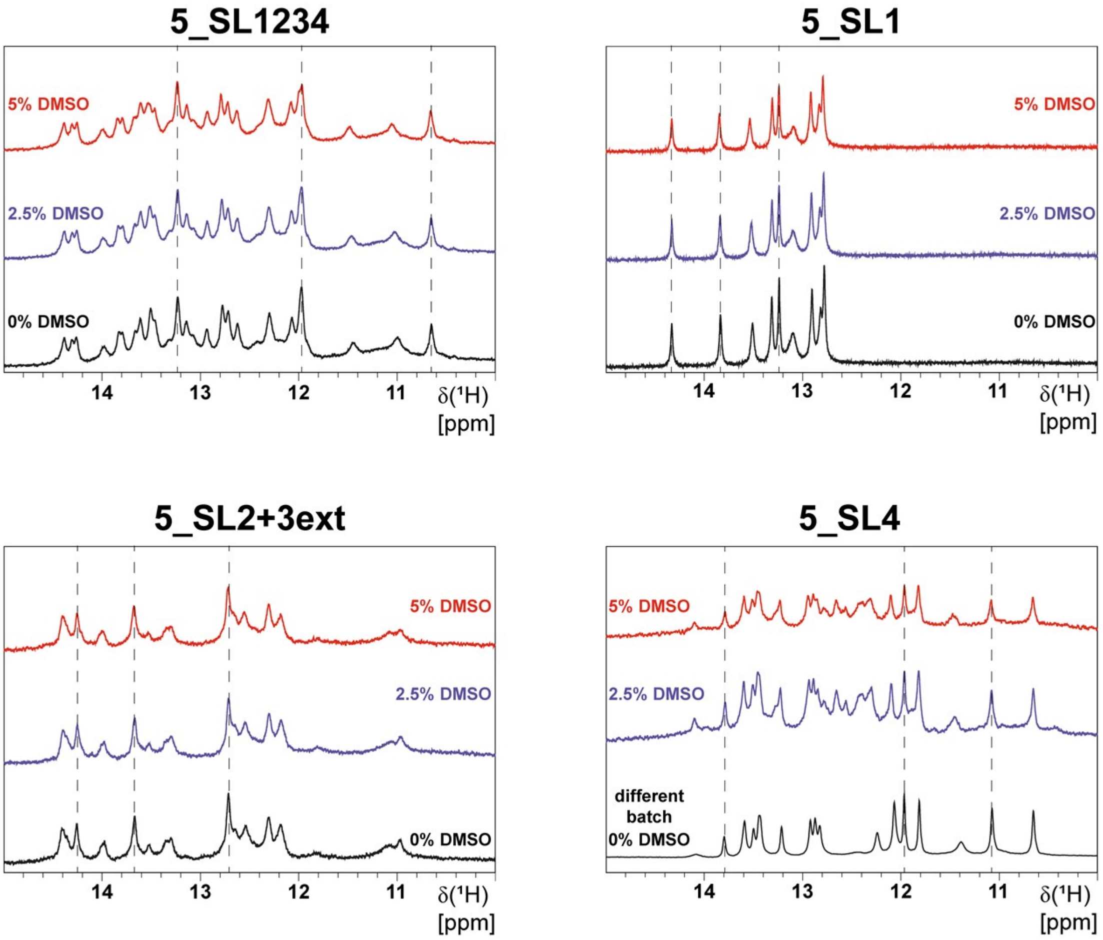

Figure 1: DMSO titrations with all used RNA constructs.

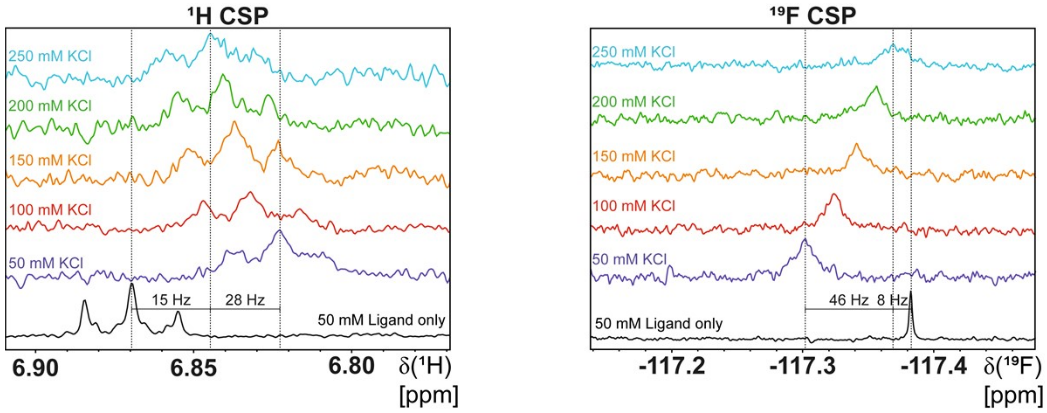

Figure 2: Effect of the salt concentration on the CSP. The spectrum in black shows the measurement of the Ligand DRTL A04 with 50 mM KCl in absence of RNA 5_SL2+3^{ext}. The other spectra represent measurements with increasing salt concentration and a Ligand-RNA ratio of 1:1.

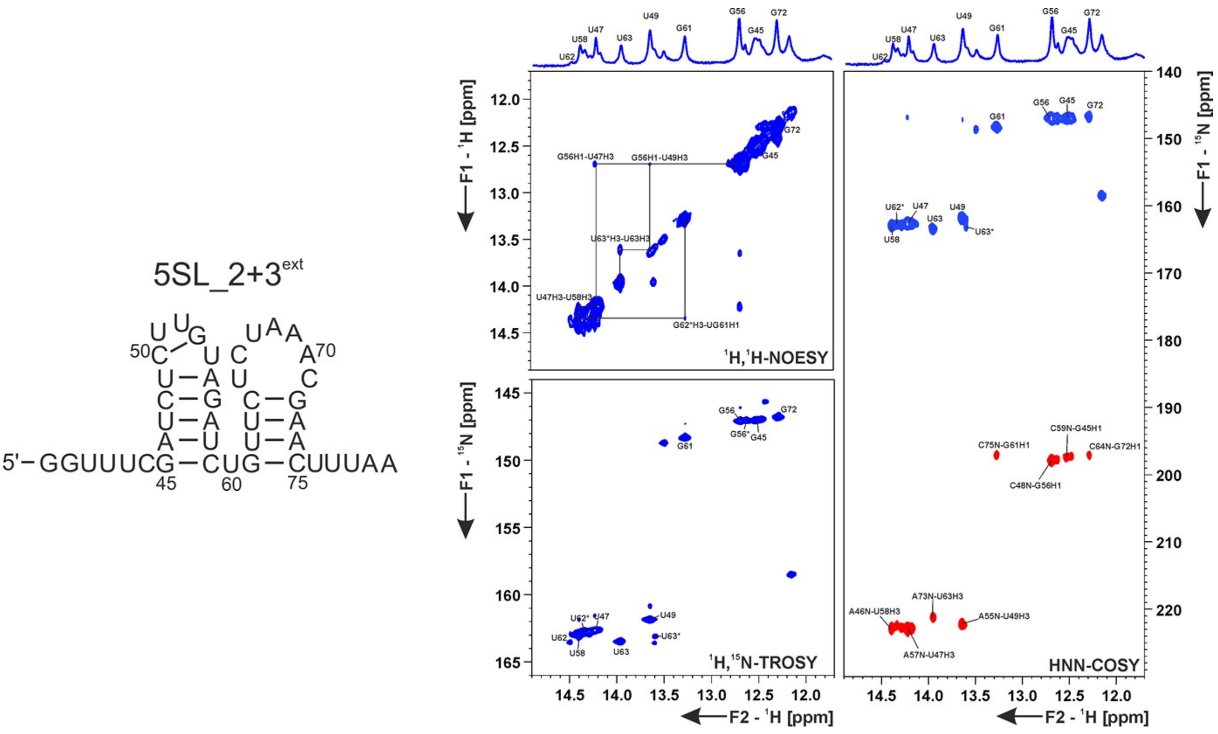

Figure 3: Secondary structure of 5_SL2+3^{ext} and assignment of the resonances of its imino region using ^{1}H-^{1}H-NOESY, ^{1}H-^{15}N-BEST-TROSY and HNN-COSY experiments. The resonances marked with a star belong to a second confirmation of the RNA sub-element.
